# Host–Guest Synergy of Metal–Organic Frameworks for Enhanced Near-Infrared Ultrafast Laser Responsiveness

**DOI:** 10.1021/acscentsci.5c00022

**Published:** 2025-03-18

**Authors:** Ruibing Lv, Lei Sun, Zhenghang Luo, Yujie Song, Shuo Li, Qi Zhang

**Affiliations:** † Institute of Chemical Materials, China Academy of Engineering Physics (CAEP), Mianyang 621900, P. R. China; ‡ School of Chemical Engineering, Chongqing University of Technology, Chongqing, 400054, P. R. China

## Abstract

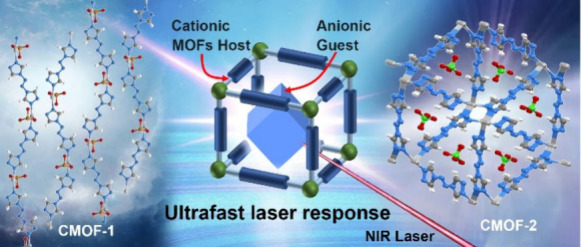

Host–guest metal–organic frameworks (MOFs) offer significant potential and value in regulating and optimizing novel material properties and functionalities, owing to the synergistic effects between the host framework and the guest units. This study reported two silver-based host–guest MOFs, [Ag(ATRZ)(BrO_3_)]_n_ (CMOF-1) and [Ag(ATRZ)_1.5_(ClO_4_)]_n_ (CMOF-2), as promising candidates for laser-responsive materials. These materials feature 1D and 3D structures, respectively, comprising Ag-ATRZ cationic MOF frameworks integrated with two distinct oxidizing anionic guests, BrO_3_^–^ and ClO_4_^–^. CMOF-1 and CMOF-2 are synthesized through straightforward, environmentally benign methods, enabling rapid fabrication. The exceptional near-infrared (NIR) laser responsiveness of CMOF-1 and CMOF-2 was achieved through the modulation of the cationic MOFs (CMOFs) architectures and synergistic interactions between the host and guest components. Moreover, both exhibit ultrafast deflagration-to-detonation transition (DDT) capabilities, alongside excellent thermal stability. This work expands the application scope of host–guest MOFs, and provides an effective strategy for developing high-performance laser-responsive materials.

## Abstract 2

This study reports Ag-based host−guest MOFs with ultrafast NIR laser responsiveness: CMOF-1 and CMOF-2, which exhibit excellent ultrafast deflagration-to-detonation transition and thermal stability.

## Introduction

**Scheme 1:**
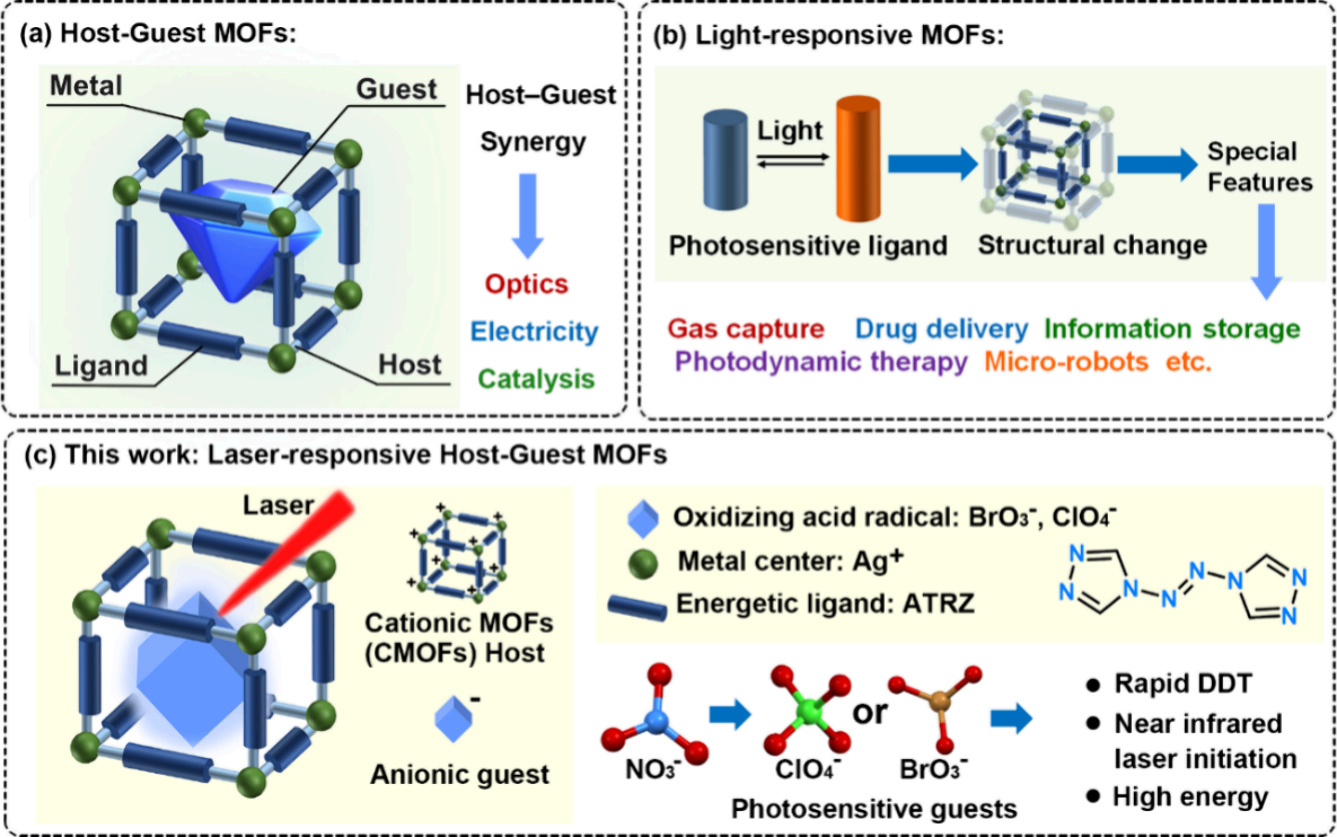
(a) Schematic Diagram of Host–Guest MOFs Structure, (b) Functional Mechanisms of Photoresponsive MOFs, (c) Ultrafast Laser Response of Cationic MOFs based on Host–Guest Synergy and Laser-Sensitive Guests

Metal–organic frameworks (MOFs) are crystalline materials characterized by ordered pores and channels, formed through the coordination bonding between metal centers and organic linkers.^1−4^ Typically synthesized via the self-assembly of metal ions or metal clusters with organic ligands, MOFs exhibit remarkable compositional diversity, structural flexibility, and tunable porosity.^5−7^ These attributes make MOFs highly versatile platforms for the orderly dispersion and stabilization of various guest molecules, such as optoelectronic enhancement units, catalysts, and pharmaceuticals, etc. (Scheme 1a) Strategic modifications to the MOF framework can alter structural rigidity and pore environments, facilitating the directed assembly of host–guest systems. These adaptations often induce distinct physical and chemical phenomena, such as electron or energy transfer, redox reactions, or synergistic host–guest interactions, which can generate functionalities beyond those of the individual components.^8,9^ For instance, incorporating fluorescent or phosphorescent guest molecules within MOFs can tune optical properties through host–guest synergy.^10−12^ Similarly, embedding electron or ion-conducting guests into MOFs can modulate conductivity by influencing charge transfer dynamics.^13−15^ Loading guest entities such as metal nanoparticles (MNPs), molecular catalysts, or enzymes into MOFs can yield advanced materials suitable for CO_2_ conversion,^16−18^ hydrogenation reactions,^19,20^ biocatalysis,^21−23^ and photocatalysis or electrocatalysis.^24−27^ Consequently, MOFs can be used as a multifunctional platform and granted new application scenarios by changing the guest molecules in the pores, which will further expand the application horizons of host–guest MOFs as high-performance materials across diverse fields.

Photoresponsive MOFs represent a burgeoning research frontier, offering tunable platforms for high-performance materials in the fields of energy, medicine and information and so on.^28,29^ As shown in Scheme 1b, these materials are constructed by integrating photoresponsive linkers or guest molecules into the MOFs structure. Upon light stimulation, the intrinsic structures and morphologies of photoresponsive MOFs can undergo reversible or irreversible transformations. These changes can enable functions such as gas adsorption and release,^30,31^ optical information storage,^32,33^ photoelectronic devices,^34,35^ and photoresponsive actuators.^36,37^ Noteworthily, light stimuli offer distinct advantages, including noncontact activation, high spatial and temporal precision, and ease of control compared to electromagnetic, thermal, or mechanical stimuli.^28^ As a result, photoresponsive MOFs hold immense potential for existing applications and provide new insights for exploring novel technological avenues.

As special light-responsive materials, laser-responsive materials are designed to convert light energy into heat or photonic resonance,^38,39^ inducing molecular bond dissociation under laser irradiation at specific wavelengths. Then a cascade of radical reactions was triggered, facilitating rapid decomposition and a deflagration-to-detonation transition (DDT).^40,41^ Such materials form the cornerstone of safe laser initiation technologies employed in the aerospace, defense, and civil blasting sectors.^42^ Compared to conventional initiation methods involving electricity, heat, or mechanical impact, laser initiation offers superior resistance to electromagnetic pulses, microwaves, static discharge, and corrosion.^43,44^ Consequently, advancing research into laser-responsive materials remains a critical and urgent endeavor. In recent years, significant progress has been made in developing ultrafast laser-responsive materials, including modifications to existing explosives and the synthesis of energetic coordination compounds.^43−47^ Despite promising advances, challenges persist in preparing a comprehensive material that exhibits exceptional energy density, stability, and initiation performance.^48,49^ Notably, there is a pressing need for near-infrared (NIR) laser-responsive materials with excellent properties.^39,45^ This demand arises because NIR laser sources, characterized by their compact size and low cost, are highly suitable for miniaturized laser ignition devices. As a result, they have garnered significant attention in the field of laser initiation of energetic materials.^50^ Inspired by the design concept of light-responsive MOFs, given the orderly holes and adjustable host frameworks, and the synergistic effect between guest units and the host skeletons, it is expected to break through the limitations of existing laser responsive materials and obtain reliable materials with near-infrared laser response.

Based on this, constructing MOF host frameworks using silver ions (Ag^+^) and the nitrogen-rich conjugated energetic ligand 4,4’-azo-1,2,4-triazole (ATRZ)^51^ was proposed, which ensures both the energetic performance and stability of the resulting materials (Scheme 1c). This approach aims to improve oxygen balance and uniformly distribute photosensitive radical generation sites within the framework by incorporating oxidizing counterion guests in a controlled, ordered manner. This crucial assembly facilitates rapid redox reactions under laser stimulation, minimizing reaction delay and achieving ultrafast laser responsiveness. Additionally, careful selection of laser-sensitive guest units can lower the initiation threshold and enhance wavelength selectivity, enabling effective near-infrared laser initiation.

Herein, two oxygen-rich anions: ClO_4_^–^ and BrO_3_^–^, were integrated as guest units into Ag-ATRZ cationic MOFs (CMOFs) hosts with high detonation heats (Scheme 1c). Two solvent-free silver-based CMOFs: [Ag(ATRZ)(BrO_3_)]_n_ (**CMOF-1**) and [Ag(ATRZ)_1.5_(ClO_4_)]_n_ (**CMOF-2**) were obtained through this approach. Comprehensive studies of their thermal stability, mechanical sensitivity, energetic performance, and laser sensitivity properties revealed that **CMOF-1** and **CMOF-2** exhibit excellent thermal stability and outstanding detonation characteristics. Importantly, both materials demonstrated efficient near-infrared laser initiation with low energy input and minimal delay periods. To better illustrate the advantages of host–guest synergy, the previously reported [Ag(ATRZ)_1.5_(NO_3_)]_n_ (**CMOF-0**),^52^ incorporating NO_3_^–^ as the guest unit, was selected as a reference material. Notably, **CMOF-0** exhibits negligible laser responsiveness. These findings confirm that the laser-responsive behavior of CMOFs can be finely tuned by adjusting oxygen-rich anionic guests, thereby providing a new approach for the development of high-performance laser-responsive materials.

## Results and Discussion

### Synthesis and Single-Crystal Structures

**Scheme 2:**
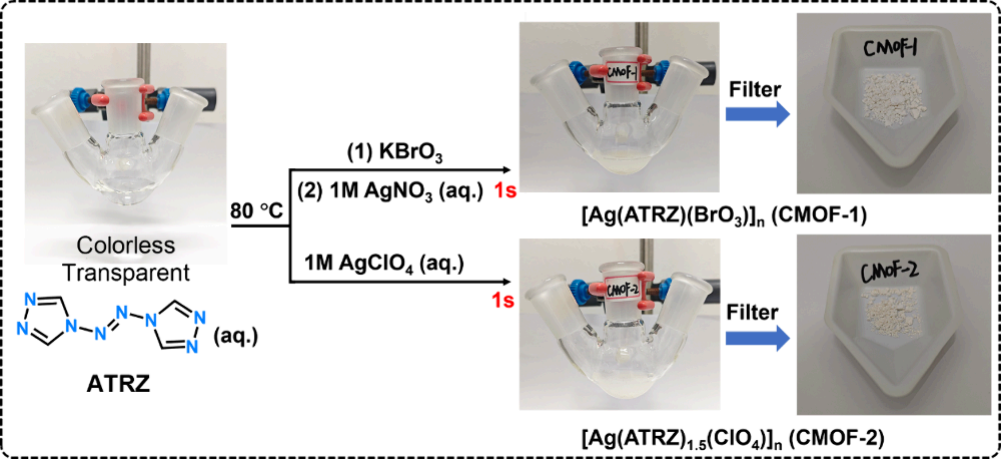
Synthesis Process of **CMOF-1** and **CMOF-2**

4,4′-Azo-1,2,4-triazole (ATRZ) was synthesized based on existing literature.^51^ As shown in Scheme 2, the “one-pot” synthesis method for **CMOF-1** and **CMOF-2** is straightforward, cost-effective, and environmentally friendly. ATRZ and inorganic salts were added to hot water successively, and the white product precipitated instantly. After filtration and air drying, pure product crystals can be quickly obtained with high yields (CMOF-1:59.5%; CMOF-2:72.5%), and the mother liquor is colorless and transparent. This synthesis process uses water as a solvent, eliminating the need for organic solvents and averting the production of polluting gases and highly toxic waste liquids, which aligns with the principles of green chemistry.

**Figure 1:**
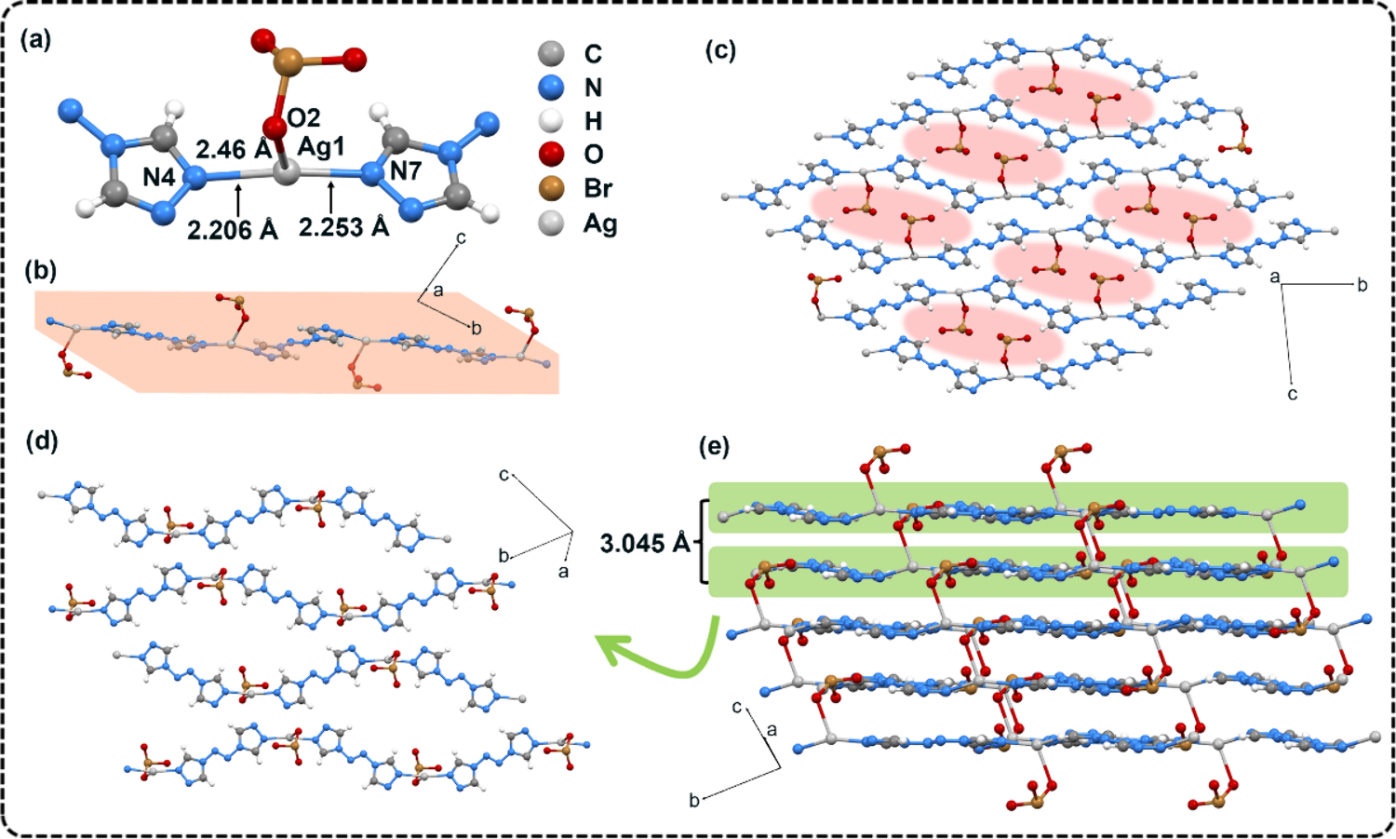
(a) Asymmetric unit of **CMOF-1** and the coordination environment between ATRZ ligand, BrO_3_^–^ guest and Ag(I) cation. (b) 1D chain-like structure of **CMOF-1**. (c) Distribution of BrO_3_^–^ guest in the 1D structure of **CMOF-1**, and the red shading indicates the pores in the structure. (d) The molecular arrangement in the same layer of **CMOF-1**. (d) The layered-like crystal packing of **CMOF-1**.

Single crystals of **CMOF-1** and **CMOF-2** suitable for characterization were prepared by recrystallization in a hot aqueous solution. Single-crystal X-ray diffraction (SCXRD) determination shows that **CMOF-1** (CCDC 2383631) crystallized in the triclinic P-1 space group, with two asymmetric units per unit cell (*Z* = 2). The optical image of **CMOF-1** shows a colorless needle-like morphology with a regular shape. Remarkably, the calculated crystal density at 296 K was as high as 2.634 g cm^–3^. As shown in Figure 1a, the asymmetric unit consists of an Ag(I) cation, an ATRZ ligand, and a BrO_3_^–^ guest anion. Each ATRZ molecule interacts with two adjacent Ag(I) ions, forming a one-dimensional (1D) chain host with the lengths of the coordination bonds Ag1–N4, Ag1–N7 were 2.206 Å and 2.253 Å, and the bond angle of N4–Ag1–N7 is 159.4°. Each central Ag^+^ is coordinated with three neighboring atoms including two N atoms and one O atom, and the O atom comes from the BrO_3_^–^ guest involved in the coordination, the bond length of Ag1–O2 was 2.46 Å. Due to the planar properties of the ATRZ molecule, all atoms in each 1D Ag-ATRZ chain are in almost the same plane (Figure 1b). Observed from the *a*-axis direction, the host chains are regularly arranged along the (0,1,-1) direction to form a plane (Figure 1d), and form a tightly stacked layered structure with a distance of 3.045 Å between each layer (Figure 1e), which contributes to the high density of CMOFs. The oxidant BrO_3_^–^ guest is anchored between the 1D host chains through coordination bonds, and fills the gaps formed by Ag-ATRZ stacking along the *a*-axis direction (Figure 1c), which is more conducive to achieving rapid redox reactions and producing a faster DDT process.

**Figure 2:**
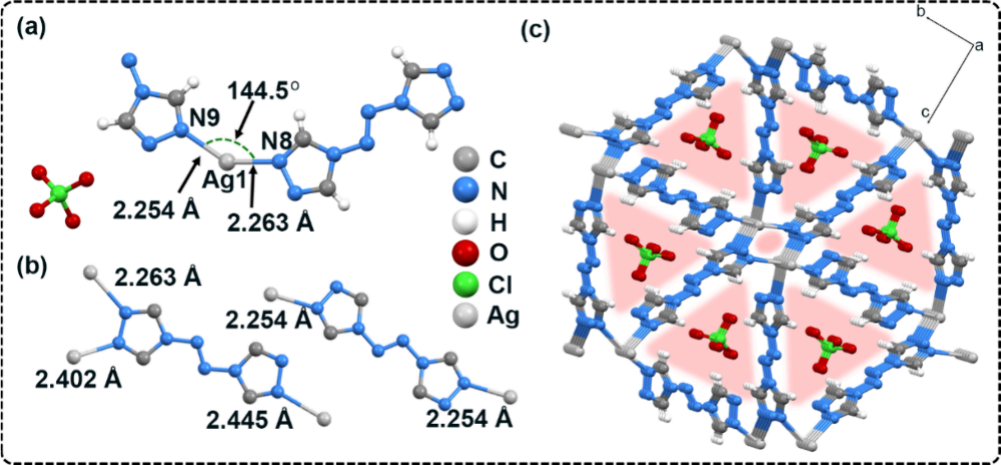
(a) Asymmetric unit of **CMOF-2**. (b) Two coordination environments between ATRZ ligand and Ag(I) cation. (c) 3D porous crystal structure of **CMOF-2**, and the red shading indicates the pores in the 3D structure.

**CMOF-2** (CCDC 2383633) crystallized in the triclinic P 21/n space group, with two asymmetric units per unit cell (*Z* = 2), and its optical image shows a colorless needle-like morphology with a regular shape. Notably, the calculated crystal density at 223 K was as high as 2.155 g cm^–3^. **CMOF-2** has a 3D irregular porous structure similar **CMOF-0**.^52^ As shown in Figure 2a, the asymmetric unit consists of an Ag(I) cation, one and a half ATRZ ligand molecules and one free ClO_4_^–^ guest anion. Each silver atom is tetracoordinated with four nitrogen atoms from ATRZ to form an irregular tetrahedral geometry. As shown in Figure 2b, different from **CMOF-1**, the coordination mode of ATRZ is divided into two types in **CMOF-2**: bidentate and tridentate. The Ag–N coordination bond length is 2.254 Å −2.445 Å, longer than the Ag–N bond in **CMOF-1**. In the 3D structure of **CMOF-2**, there are two different channels along the *a*-axis. The first is a channel with a larger pore size, which is composed of three ATRZ molecules and Ag(I) ions as linkers. The oxidant guest ClO_4_^–^ is free in the channel, which is different from the BrO_3_^–^ involved in the coordination in **CMOF-1**. This allows for uniform distribution and full contact in the host skeleton, which is also conducive to the occurrence of a rapid DDT process under laser stimulation.

### Stability and Sensitivity

**Figure 3:**
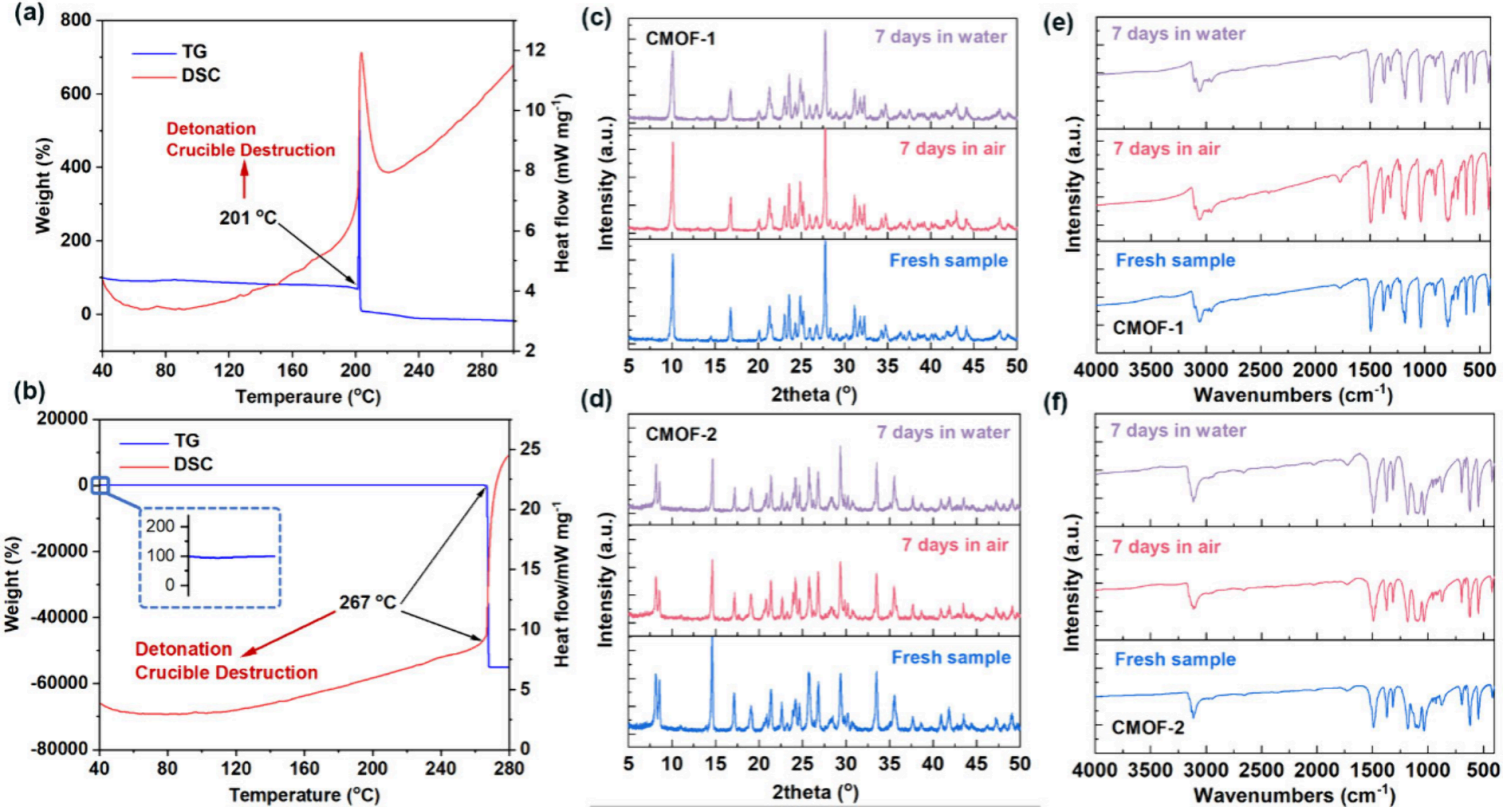
(a) TG-DSC curves of **CMOF-1**. (b) TG-DSC curves of **CMOF-2**. (c) Stability testing of **CMOF-1** by PXRD. (d) Stability testing of **CMOF-2** by PXRD. (e) Stability testing of **CMOF-1** by FTIR. (d) Stability testing of **CMOF-2** by FTIR.

Heat resistance is a key factor in determining whether laser-responsive materials can function reliably in high-temperature environments. The thermal stabilities of **CMOF-1** and **CMOF-2** were investigated through thermogravimetry (TG) and differential scanning calorimetry (DSC) at a heating rate of 10 K min^–1^ under an argon atmosphere. The TG-DSC curves of **CMOF-1** and **CMOF-2** were shown in Figure 3a and Figure 3b, respectively. Both did not show melting and phase change during the early programmed temperature increase, but exhibited a rapid one-step explosive decomposition process characterized by sharp exothermic peaks. Their explosive decomposition severely damaged the ceramic crucibles, even though the initial mass of the **CMOF-1** and **CMOF-2** samples was only 0.114 mg and 0.069 mg, respectively. These phenomena indicate their ultrafast DDT properties under thermal loading. Additionally, the thermal decomposition temperature (*T*_d_) of **CMOF-1** is 201 °C, which is almost the same as that of copper azide (CA) (*T*_d_ = 205 °C). The thermal decomposition temperature of **CMOF-2** is 267 °C, which aligns closely with the *T*_d_ value of the classic laser-responsive material [Co(NH_3_)_4_(NT)]ClO_4_ (BNCP) (*T*_d_ = 268 °C) and higher than **CMOF-1**, this could be attributed to the stable 3D CMOF host. Moreover, the thermal decomposition temperature of **CMOF-2** is 37 °C higher than that of [Ag(ATCA)ClO_4_]_n_,^53^ another laser-responsive material synthesized by our group, which serves as a representative of Ag-based laser-sensitive primary explosives. In addition, both **CMOF-1** and **CMOF-2** demonstrate superior thermal stability compared to most organic primary explosives, including DDNP (*T*_d_ = 157 °C), making them suitable for various military and civilian applications. Noteworthily, **CMOF-1** and **CMOF-2** show remarkable insensitivity to both air and water. This conclusion is supported by powder X-ray diffraction (PXRD) and Fourier Transform Infrared Spectrometer (FTIR) analyses conducted over a period of 7 days (Figure 3c-3f), all samples were from the same batch of products and must be dried identically (80 °C, 3 h) before PXRD and FTIR testing to minimize moisture content. The results showed that both materials meet the environmental stability standards typically required for laser-responsive materials.

The mechanical sensitivities of **CMOF-1** and **CMOF-2** were evaluated experimentally in accordance with the BAM standard procedures. Impact sensitivity (IS) was measured using the standard BAM Fall Hammer, while friction sensitivity (FS) was assessed using the BAM friction tester. The results are summarized in Table 1. Both **CMOF-1** and **CMOF-2** exhibit mechanical sensitivities typical of primary explosives (**CMOF-1**: IS = 0.75 J, FS < 5 N; **CMOF-2**: IS = 0.5 J, FS = 5 N), a characteristic attributed to the incorporation of oxygen-rich guest molecules. In comparison, the impact sensitivity of **CMOF-0** (IS = 30 J)^52^ is significantly lower, and the sensitivity ranking of the three materials following the order: NO_3_^–^ < ClO_4_^–^ < BrO_3_^–^. This optimized sensitivity ensures that the CMOFs can be effectively initiated when employed as laser-responsive materials.

**Table 1:** Comparison of the Physical and Energetic Properties of **CMOF-1**, **CMOF-2** ,and Other Selected Compounds^a^

Comp.	*N*^b^ [%]	ρ^c^ [g cm^–3^]	*T*_d_^d^ [°C]	OB_CO_^e^ [%]	OB_CO2_^f^ [%]	IS^g^ [J]	FS^h^ [N]	λ^i^ [nm]	*t*^j^ [ms]	*E*^k^ [mJ]
**CMOF-1**	28.02	2.634	201	–14.0	–30.0	0.75	<5	808	1.14	11.4
**CMOF-2**	30.15	2.155^l^	267	–19.4	–40.6	0.5	5	808	0.15	1.5
**CMOF-0**	43.76	3.160	257	–25.1	–48.2	30	-	808	X	X
ATRZ	68.3	1.620	313	–58.5	–97.5	14	160	808	X	X
[Ag(ATCA)(ClO_4_)]_*n*_^m^	24.00	2.534	230	–4.6	–18.3	5	72	800	72.68	207
[Cu(N_3_)_2_(1-NET)]^n^	50.24	1.985	122	–18.3	–34.0	3	1	915	-	25.5
[Pb(OH-ATZ)_2_]_n_^o^	34.4	3.180	284	–11.8	–19.7	10	50	800	5.22	27.0
CA^p^	56.90	2.600	205	–10.9	–10.9	<1	≤0.1	-	-	-
LA^p^	28.90	4.800	315	–5.5	–5.5	2.5–4	0.1–1	-	-	-
DDNP^p^	26.70	1.720	157	–15.2	–60.9	<1	24.7	-	-	-

a “-” indicates that no data was obtained, and “X” indicates that it cannot be initiated by the laser.

b Nitrogen content.

c Density.

d Decomposition temperature.

e Oxygen balance based on CO.

f Oxygen balance based on CO_2_.

g Impact sensitivity.

h Friction sensitivity.

i Laser wavelength.

j Laser initiation delay time.

k Laser initiation energy value.

l Crystal density (223 K).

m Reference (53).

n Reference (58).

o Reference (54).

p Reference (59).

### Modified Koenen Test

**Figure 4:**
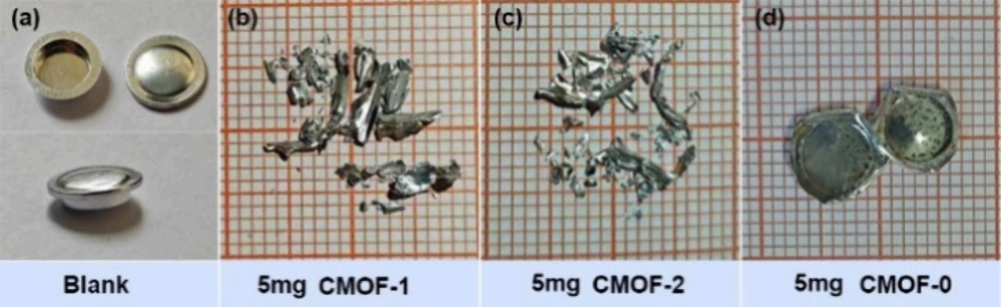
Modified Koenen test of **CMOF-1**, **CMOF-2**, and **CMOF-0**.

To experimentally examine the impact of NO_3_^–^, BrO_3_^–^, and ClO_4_^–^ on the explosive properties of laser-responsive materials, a modified Koenen test was conducted, using a Bunsen burner for rapid heating. This approach served to characterize the DDT process of **CMOF-1**, **CMOF-2**, and [Ag(ATRZ)_1.5_(NO_3_)]_n_, with the experimental results shown in Figure 4. For each test, a precisely weighed 5 mg sample of loose powder was sealed within an aluminum crucible and subjected to rapid heating. After combustion or detonation, the crucible fragments were collected for a preliminary evaluation of the explosive performance of the materials. The results demonstrated that **CMOF-1** and **CMOF-2**, which incorporate BrO_3_^–^ and ClO_4_^–^ as guest units, respectively, exhibit exceptional explosive characteristics. Similar to the typical primary explosives lead azide (LA), the brief heating triggered a rapid deflagration-to-detonation transition (DDT) of **CMOF-1** and **CMOF-2**, resulting in explosions that caused significant damage to the crucibles (Figure 4b and Figure 4c). In contrast, **CMOF-0**, which incorporates a NO_3_^–^ guest unit, underwent rapid decomposition under intense heating, leading to only minor deformation of the crucible without any rupture (Figure 4d). In brief, among the tested oxidizing guest units, both BrO_3_^–^ and ClO_4_^–^ were more effective in facilitating detonation than NO_3_^–^, as evidenced by their faster transition to detonation. These findings underscore the critical role of oxidizing guest molecules in modulating the DDT process of laser-responsive materials under thermal stress, highlighting the influence of specific guest species on detonation efficiency.

### Laser-Response Performance

**Figure 5:**
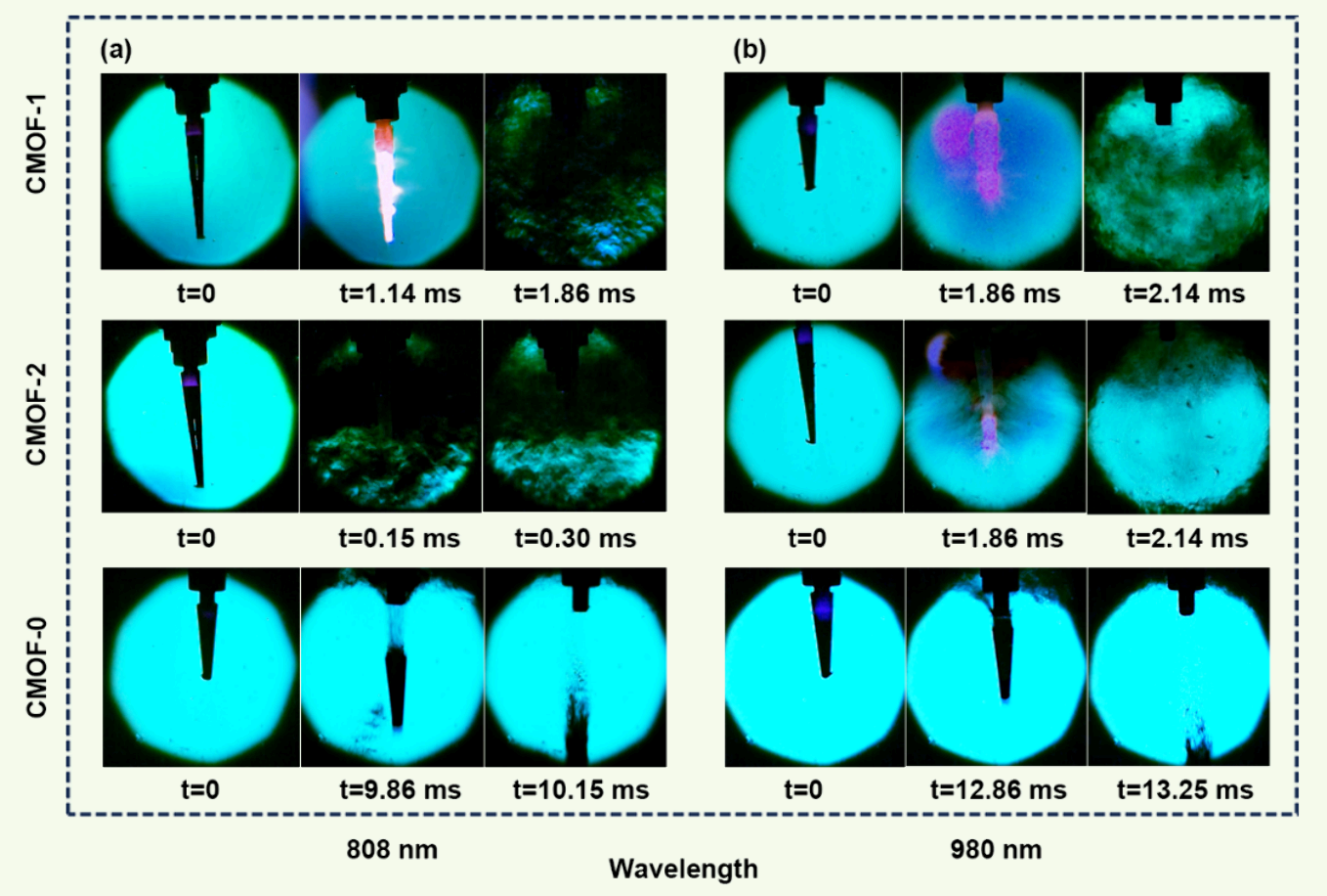
(a) 808 nm (10 W) laser response process of **CMOF-1**, **CMOF-2,** and **CMOF-0**. (b) 980 nm (10 W) laser response process of **CMOF-1**, **CMOF-2,** and **CMOF-0**.

Based on the excellent fast deflagration-detonation transition (DDT) performance of **CMOF-1** and **CMOF-2**, six sets of parallel experiments were conducted to further evaluate their laser-responsive properties at two distinct wavelengths (808 and 980 nm). These tests were compared against **CMOF-0**, a CMOF with nitrate (NO_3_^–^) as the guest molecule. All experiments were carried out under identical conditions, maintaining consistent sample weight and laser parameters (wavelength and power). High-speed cameras captured the response of the three CMOFs under laser irradiation at near-infrared wavelengths of 808 and 980 nm, as shown in Figure 5a and Figure 5b. Both **CMOF-1** and **CMOF-2** demonstrated rapid and violent explosions shortly after brief exposure to either 808 or 980 nm laser irradiation, resulting in significant damage to the confinement tube. These detonations were accompanied by intense acoustic emissions, and **CMOF-1** produced a bright flame column within the transparent tube. In stark contrast, the laser responsiveness of **CMOF-0** is significantly weaker. with only partial ignition observed and no substantial structural damage to the tube. These results align with findings from the Modified Koenen test. To quantitatively assess the laser-responsive capabilities of these compounds, the initiation delay time (*t*) and initiation energy (*E*) were used as evaluation metrics. As shown in Figure 5a, **CMOF-2** displayed superior laser-responsive performance (*t* = 0.15 ms, *E* = 1.5 mJ) compared to **CMOF-1** (*t* = 1.14 ms, *E* = 11.4 mJ) when irradiated with 808 nm laser, outperforming most existing laser-responsive materials, e.g. [Ag(ATCA)(ClO_4_)]_n_ (*t* = 72.68 ms, *E* = 207 mJ),^53^ [Pb(OH-ATZ)_2_]_n_ (*t* = 5.22 ms, *E* = 27 mJ),^54^ and [Cu(PZCA)_2_(ClO_4_)_2_] (*t* = 5.0 ms, *E* = 45 mJ),^41^ this phenomenon is related to the host–guest synergy effects present in **CMOF-1** and **CMOF-2**. Under laser irradiation, the halogen-oxygen bonds within the guest units undergo cleavage, producing a large amount of highly reactive oxygen radicals. These radicals subsequently initiate chain reactions by attacking the host framework, thereby facilitating the rapid and efficient decomposition of the CMOFs. When subjected to 980 nm laser irradiation, both **CMOF-1** and **CMOF-2** exhibited slightly longer initiation delay times of 1.86 ms (Figure 5b), with corresponding initiation energies of 18.6 mJ. This variation may be attributed to the dependence of the light absorption capacity and photothermal conversion efficiency of CMOFs on the laser wavelength, which influences the rate of energy absorption and initiation.^50,55,56^ These findings demonstrate that **CMOF-1** and **CMOF-2** exhibit exceptional laser responsiveness within the shortwave near-infrared (SW-NIR) range, aligning perfectly with our design principles, and underscore the remarkable potential of host–guest CMOF-based materials for high-efficiency, near-infrared laser response applications, highlighting their robust and tunable performance characteristics.

### Theoretical Investigation of Ignition Mechanism

It is generally accepted that the laser response under infrared irradiation is primarily driven by thermal loading resulting from photothermal conversion. The decomposition of laser-responsive energetic materials is typically initiated by highly reactive oxygen radicals generated from oxidizing anions due to photothermal effects.^41^ The complexity of this process directly reflects the laser response capability of materials. In this study, we employed Gaussian16 (Revision A. 03) software,^57^ using density functional theory (DFT) to calculate both the dissociation energy of the weakest bonds in the ligands and the bond dissociation energies (BDEs) required to generate oxygen radicals (Figure 5), all geometry optimizations and energy calculations were at the theoretical level of B3LYP/6–31G* (nonmetallic elements) combined with Stuttgart/Dresden (SDD) pseudopotential (Ag). The results reveal that a set of N–N bonds in ATRZ exhibits the lowest BDE value at 175.2 kJ mol^–1^. Among the three oxidizing anions (BrO_3_^–^, ClO_4_^–^, and NO_3_^–^) studied, the BDE values required for O radical formation increase in the following order: O-ClO_3_ (282.7 kJ mol^–1^) < O-BrO_2_ (351.7 kJ mol^–1^) < O-NO_2_ (411.1 kJ mol^–1^). This indicates that ATRZ dissociation occurs most readily, followed by ClO_4_^–^ and BrO_3_^–^, with NO_3_^–^ being the most difficult to dissociate. The results align closely with laser-initiation experiments, suggesting that the laser response of **CMOF-1** and **CMOF-2** results from the synergistic interaction between host frameworks and guest anions, **CMOF-1**: [Ag^+^(ATRZ)]_n_ and BrO_3_^–^, **CMOF-2**: [Ag^+^(ATRZ)_1.5_]_n_ and ClO_4_^–^. Upon decomposition of ATRZ, guest molecules rapidly produce highly reactive oxygen radicals, accelerating the deflagration-to-detonation transition (DDT) and facilitating the reaction process. In contrast, under laser irradiation, **CMOF-0** undergoes rapid ATRZ decomposition, but the NO_3_- anion is not easily triggered, thus preventing a detonation-level laser response. Therefore, this study presents an effective strategy for enhancing near-infrared laser initiation in energetic CMOFs by modulating their response through host–guest synergistic effects.

**Figure 6:**
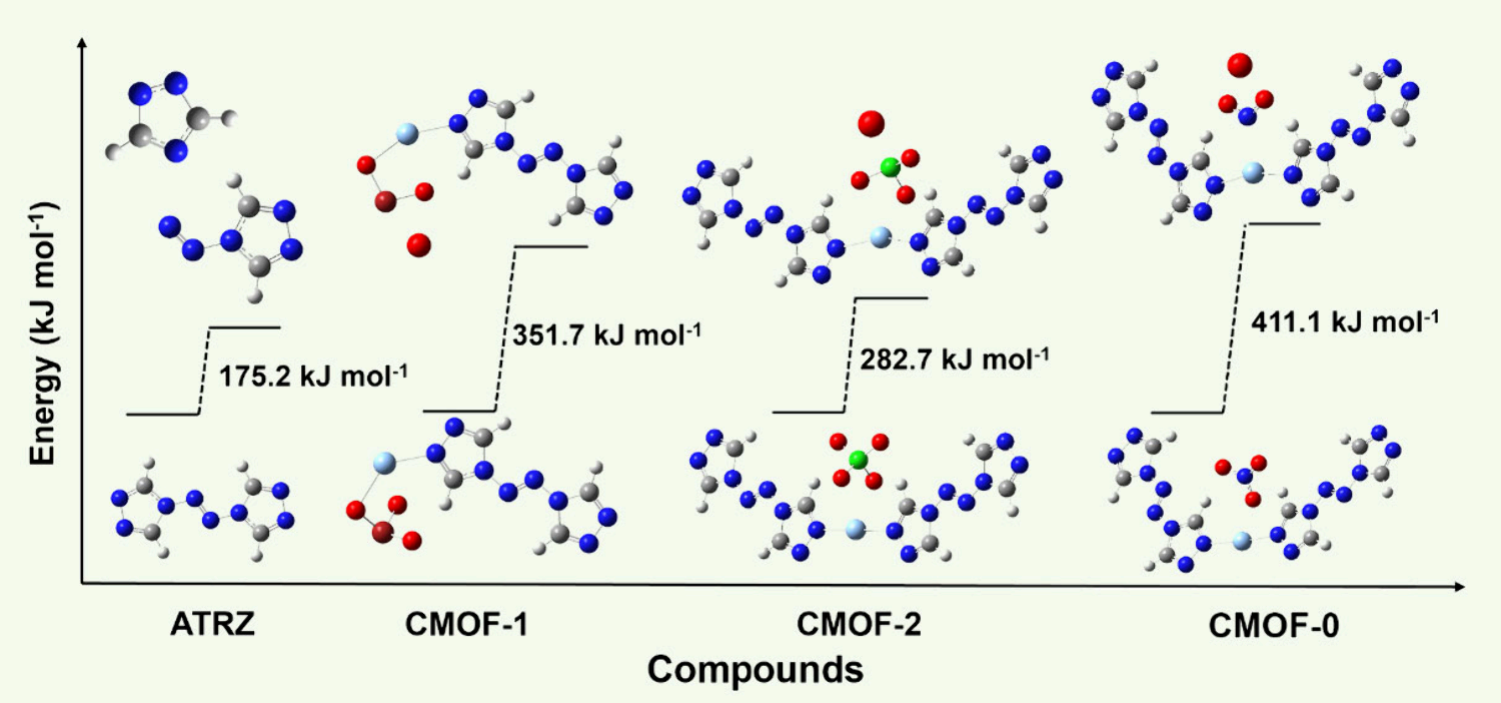
Bond dissociation energy (BDE) of ATRZ, **CMOF-1**, **CMOF-2**, and **CMOF-0**.

## Conclusion

In summary, this study has demonstrated that laser-responsive materials with superior thermal stability and rapid initiation times can be constructed through a host–guest MOF strategy. By rationally modulating the synthesis and assembly of the Ag-ATRZ cationic MOF framework and oxidizing anionic guests, it is possible to design materials with laser response capabilities. Combined with their environmentally friendly and scalable rapid self-assembly in aqueous systems, this approach enables efficient material preparation. Moreover, the host–guest CMOF structures impart **CMOF-1** and **CMOF-2** with remarkable thermal stability and exceptional deflagration-to-detonation transition (DDT) capabilities, as reflected by their ultrafast thermal decomposition at 201 and 267 °C, respectively. Most notably, through designing the host–guest MOFs architecture, the synergistic effects between the host and guest units ensure outstanding laser response properties, characterized by initiation times of 1.14 and 0.15 ms for **CMOF-1** and **CMOF-2**. The energetic Ag-ATRZ host framework offers an ideal platform for the development of novel laser-responsive materials, effectively balancing energy density and laser sensitivity attributes essential for advanced energetic applications. This strategy is anticipated to serve as a promising pathway for the rational design and fabrication of laser-responsive materials with innovative architectures, thereby expanding their potential applications.

## Supporting Information Available

Additional experimental details, materials, and methods. FTIR, PXRD curves and crystallographic data for all compounds. (PDF)
Crystallographic file for CMOF-1 (CIF)
Crystallographic file CMOF-2 173K (CIF)
Crystallographic file CMOF-2 223K (CIF)

## Supplementary Material

oc5c00022_si_001.pdf

oc5c00022_si_002.cif

oc5c00022_si_003.cif

oc5c00022_si_004.cif
