# Supplementary material for: Host–Guest Synergy of Metal–Organic Frameworks for Enhanced Near-Infrared Ultrafast Laser Responsiveness
Source: ACS Cent Sci. 2025 Mar 18;11(4):583–91. doi: 10.1021/acscentsci.5c00022 (PMC12022905; doi:10.1021/acscentsci.5c00022)
Supplement: Supplementary file 1 — oc5c00022_si_001.pdf [file oc5c00022_si_001.pdf]

Supporting Information (SI)

Host-Guest Synergy of Metal-Organic Frameworks for Enhanced Near-Infrared Ultrafast Laser Responsiveness

Ruibing Lv,[1] Lei Sun,[2] Zhenghang Luo,[1] Yujie Song,[1] Shuo Li,[2]* Qi Zhang[1]*

[1] Institute of Chemical Materials (ICM), China Academy of Engineering Physics (CAEP), Mianyang 621900, P. R. China

[2] School of Chemical Engineering, Chongqing University of Technology, Chongqing, 400054, P. R. China

* Corresponding author

* E-mail: jackzhang531@ caep.cn (Qi Zhang)

* E-mail: lishuo@cqut.edu.cn (Shuo Li)

Table of Contents

1. Experimental Section S3-S4
2. FTIR spectroscopy S4
3. PXRD spectroscopy S4
4. X-ray Crystallography S5-S13

1. Experimental Section

Caution! Although no accidental explosion occurred during the synthesis and handling, **CMOF-1** and **CMOF-2** are primary explosives, which are sensitive. Thus, appropriate safety pre-cautions should be taken in every energetic material study. It is recommended that protective clothing, latex gloves, goggles, earplugs, and masks be worn during the experiment, and all unnecessary stimulations such as fire, impact, friction, and static electricity to the energetic materials must be eliminated.

Reagents and General Methods: Acetic acid (99.5%, AR), 4-amino-1,2,4-triazole (98.0%, AR), sodium dichloroisocyanurate (96.0%), potassium bromate (99%, AR), silver nitrate (99%, AR), and silver perchlorate hydrate (99%) were purchased from Shanghai Aladdin Biochemical Technology Co., Ltd. Infrared (IR) spectra were performed on the Perkin-Elmer Spectrum II IR Spectrometers using KBr pellets, and all samples were tested after drying. The powder X-ray diffraction patterns were collected on a Bruker D8 Advance, and the 2θ range measured was 5-50° with steps of 0.02°/0.1s. The single crystal data were collected using an Oxford Diffraction Xcalibur, the structures were solved and refined with the SHELXS program in the Olex2/1.2 software suite. Thermogravimetric and differential scanning calorimetry analysis curves were recorded on a Mettler Toledo calorimeter equipped with an auto-cooling accessory at a scan rate of 10 K min^{-1}. Impact and friction sensitivity data were measured using a standard BAM Fall hammer and a BAM friction tester. The structural stability of CMOF-1 and CMOF-2 during the ageing process through a 7 day powder X-ray diffraction (PXRD) experiment and Fourier transform infrared spectroscopy (FTIR) testing. To obtain accurate results, all test samples were sourced from the same product batch, and stored in air and water in a room at a constant temperature of 26°C. Before each testing session, the samples underwent uniform drying conditions (80 °C for 3 h) to minimize moisture content.

Synthesis of 4,4'-azo-1,2,4-triazole (ATRZ): Sodium dichloroisocyanurate (20.12 g, 96 mmol) was dissolved in 200 mL of deionized water at room temperature with stirring until complete dissolution. Subsequently, 10 mL of acetic acid was added, and the mixture was vigorously stirred at room temperature for 4 hours, maintaining the reaction temperature below 10 °C using an ice bath. Separately, 4-amino-1,2,4-triazole (10.1 g, 120 mmol) was dissolved in 10 mL of water and added to the reaction mixture. Stirring was continued for an additional 5 hours to ensure completion of the reaction. The reaction mixture was then filtered, and the solid obtained was dissolved in 300 mL of deionized water by boiling. After cooling for 3 hours, the solution was filtered again to isolate needle-shaped crystals of ATRZ. Yield: 5.12 g, 52%.

Synthesis of $[Ag(ATRZ)_{1.5}(NO_3)]_n$: $[Ag(ATRZ)_{1.5}(NO_3)]_n$ was synthesized according to a method reported in the literature. 4,4'-azo-1,2,4-triazole (ATRZ) (1mmol, 0.164 g) was added to 15mL of deionized water, heated to 80 °C and stirred until dissolved. Then, AgNO3 aqueous solution (1M, 2mL) was added dropwise to the reaction system, and a large amount of white precipitate was immediately precipitated. The product was collected by filtration, washed with deionized water and ethanol, and finally dried in air.

Synthesis of $[Ag(ATRZ)(BrO_3)]_n$ (CMOF-1): 4,4'-azo-1,2,4-triazole (ATRZ) (1 mmol, 0.164 g) and KBrO3 (2 mmol, 0.334 g) were added to 15 mL of deionized water, heated to 80 °C and stirred until dissolved. Then, $AgNO_3$ aqueous solute on (1M, 3 mL) was added dropwise to the reaction system, and a large amount of white precipitate was immediately precipitated. The product was collected by filtration, washed with deionized water and ethanol, and finally dried in air to yield CMOF-1 (0.30 g, 59.5%). Anal. calcd for $C_4H_4AgBrN_8O_3$ (399.92 g mol^{-1}): C 12.01, H 1.01, N 28.02; found: C 12.11, H 1.02, N 27.98.

Synthesis of [Ag(ATRZ)$_{1.5}$(ClO4)]$_n$ (CMOF-2): 4,4'-azo-1,2,4-triazole (ATRZ) (1 mmol, 0.164 g) was added to 15mL of deionized water, heated to 80 °C and stirred until dissolved. Then, $AgClO_4$ aqueous solution (1M, 2 mL) was added dropwise to the reaction system, and a large amount of white precipitate was immediately precipitated. The product was collected by filtration, washed with deionized water and ethanol, and finally dried in air to yield CMOF-2 (0.49 g, 72.5 %). Anal. calcd for $C_6H_6AgClN_{12}O_4$ (453.55 g mol^{-1}): C 15.89, H 1.33, N 37.06; found: C 15.96, H 1.26, N 36.97.

2. FTIR spectroscopy

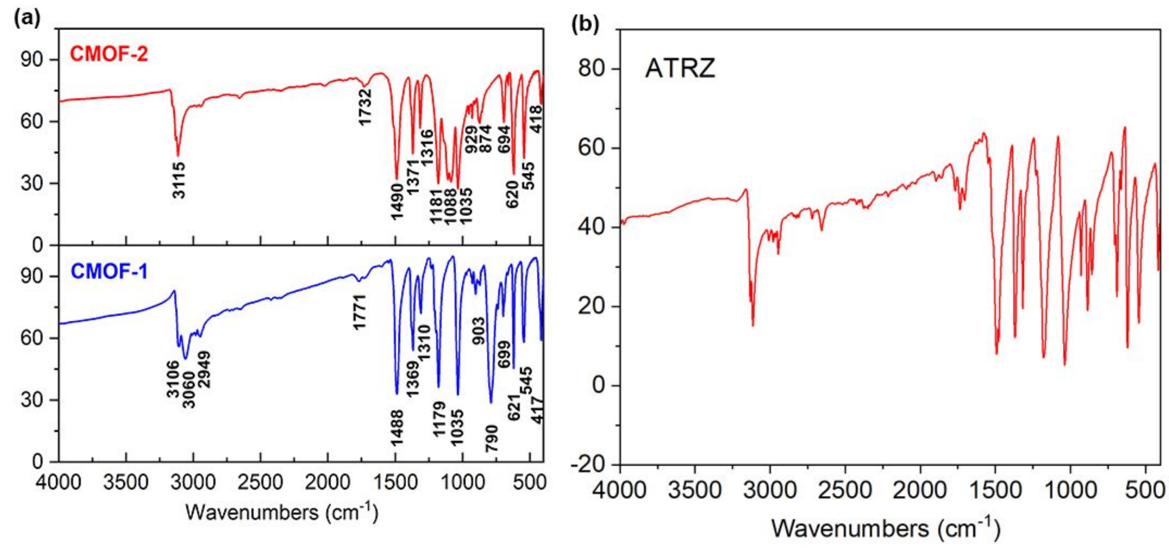

Figure S1. FTIR Curves of CMOF-1, CMOF-2, and ATRZ.

3. PXRD spectroscopy

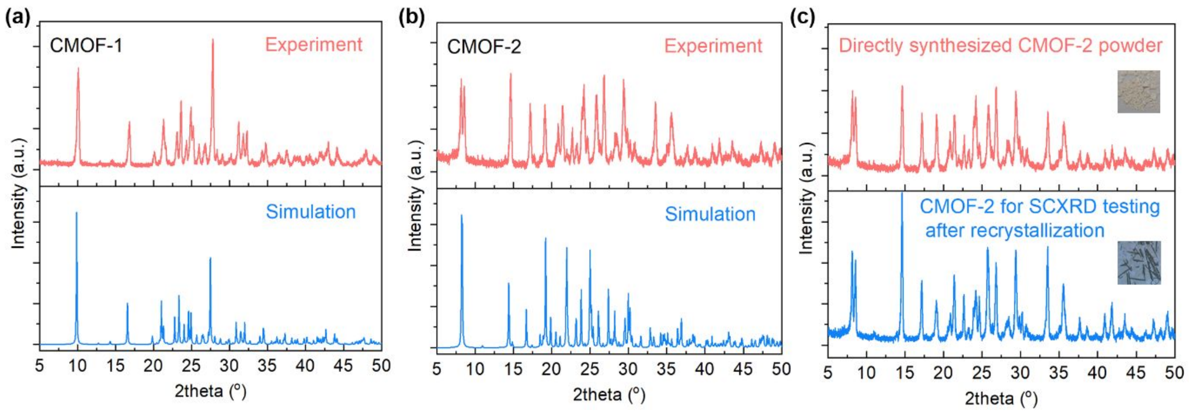

Figure S2. (a) Comparison of experimental PXRD spectra and simulated spectra of CMOF-1. (b) Comparison of experimental PXRD spectra and simulated spectra of CMOF-2 (the observed discrepancies primarily originate from the substantial temperature difference between the SCXRD data collection and the experimental PXRD tests) (c) Comparison of PXRD spectra of directly synthesized CMOF-2 powder and CMOF-2 for SCXRD testing after recrystallization.

4. X-ray Crystallography

4.1 CMOF-1

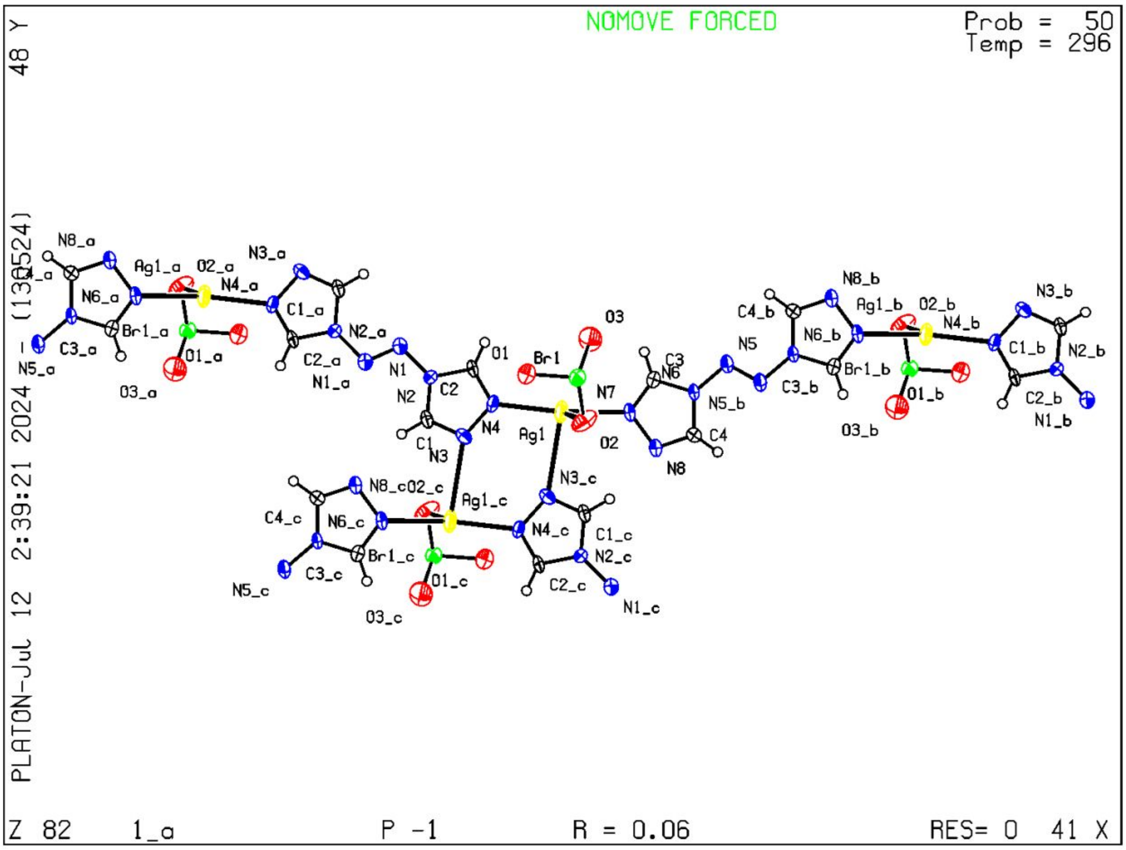

Figure S3. The crystal structure of $[Ag(ATRZ)(BrO_3)]_n$ (CMOF-1).

Table S1. Crystal data and structure refinement for $[Ag(ATRZ)(BrO_3)]_n$ (CMOF-1).

**Compounds**	$[Ag(ATRZ)(BrO_3)]_n$ (CMOF-1)
CCDC number	2383631
Empirical formula	$C_4H_4AgBrN_8O_3$
Formula weight	399.92
Temperature/K	296
Crystal system	triclinic
Space group	*P*-1
a/Å	4.5279(2)
b/Å	9.0596(5)
c/Å	12.4675(7)
α/°	84.031(2)
β/°	87.102(2)
γ/°	82.798(1)
Volume/Å^3	504.28(5)
Z	2
ρ_{calc}g/cm^3	2.634
μ/mm^{-1}	5.974

F(000)	380.0
Crystal size/mm^3	0.12 × 0.1 × 0.08
Radiation	Mo Kα (λ = 0.71073)
2Θ range for data collection/°	5.348 to 56.624
Index ranges	-6 ≤ h ≤ 6, -12 ≤ k ≤ 12, -16 ≤ l ≤ 16
Reflections collected	9516
Independent reflections	2466 [R_{int} = 0.0652, R_{siama} = 0.0848]
Data/restraints/parameters	2466/0/154
Goodness-of-fit on F^2	1.087
Final R indexes [I>=2σ (I)]	R_1 = 0.0551, WR_2 = 0.0788
Final R indexes [all data]	R_1 = 0.1085, WR_2 = 0.0963
Largest diff. peak/hole / e Å^{-3}	0.80/-0.90

Table S2. Selected bond lengths [Å] for $[Ag(ATRZ)(BrO_3)]_n$ (CMOF-1).

Atom	Atom	Length/Å	Atom	Atom	Length/Å
Ag1	N4	2.207(5)	N6	C3	1.353(8)
Ag1	N7	2.253(5)	N6	C4	1.365(8)
Ag1	O2	2.459(5)	N4	N3	1.391(7)
Br1	O1	1.634(5)	N4	C2	1.289(8)
Br1	O2	1.652(5)	N3	C1	1.292(8)
Br1	O3	1.633(5)	N7	N8	1.406(7)
N2	N1	1.386(7)	N7	C3	1.290(8)
N2	C2	1.368(7)	N5	N5^1	1.230(9)
N2	C1	1.359(8)	N1	N1^2	1.230(9)
N6	N5	1.386(7)	N8	C4	1.291(7)

Table S3. Selected bond Angles [°] for $[Ag(ATRZ)(BrO_3)]_n$ (CMOF-1).

Atom	Atom	Atom	Angle/˚	Atom	Atom	Atom	Angle/˚
N4	Ag1	N7	159.4(2)	C2	N4	N3	108.7(5)
N4	Ag1	O2	113.02(19)	C1	N3	N4	106.8(5)
N7	Ag1	O2	87.16(19)	N8	N7	Ag1	124.7(4)
O1	Br1	O2	104.0(3)	C3	N7	Ag1	123.5(4)
O3	Br1	O1	104.8(3)	C3	N7	N8	107.6(5)
O3	Br1	O2	105.7(3)	N51	N5	N6	110.5(6)
C2	N2	N1	123.2(5)	N12	N1	N2	109.7(6)
C1	N2	N1	130.6(5)	C4	N8	N7	107.6(5)
C1	N2	C2	106.1(5)	N7	C3	N6	109.2(6)
C3	N6	N5	122.5(5)	N8	C4	N6	108.7(6)
C3	N6	C4	107.0(5)	N4	C2	N2	108.5(6)
C4	N6	N5	130.5(5)	N3	C1	N2	109.8(5)
N3	N4	Ag1	124.3(4)	Br1	O2	Ag1	111.8(2)
C2	N4	Ag1	126.7(4)				

Table S4. Selected torsion Angles [°] for $[Ag(ATRZ)(BrO_3)]_n$ (CMOF-1).

Atom	Atom	Atom	Atom	Angle/°	Atom	Atom	Atom	Atom	Angle/°
Ag1	N4	N3	C1	174.9(4)	C3	N6	N5	N51	172.1(7)
Ag1	N4	C2	N2	-174.8(4)	C3	N6	C4	N8	-0.2(8)
Ag1	N7	N8	C4	-157.3(5)	C3	N7	N8	C4	0.0(7)
Ag1	N7	C3	N6	157.5(4)	C4	N6	N5	N51	-9.5(11)
N4	N3	C1	N2	0.0(7)	C4	N6	C3	N7	0.2(8)
N3	N4	C2	N2	-0.2(7)	C2	N2	N1	N12	-173.7(7)
N7	N8	C4	N6	0.1(7)	C2	N2	C1	N3	-0.1(8)
N5	N6	C3	N7	178.9(6)	C2	N4	N3	C1	0.2(7)
N5	N6	C4	N8	-178.8(6)	C1	N2	N1	N12	4.2(11)
N1	N2	C2	N4	178.5(6)	C1	N2	C2	N4	0.2(8)
N1	N2	C1	N3	-178.3(6)	O1	Br1	O2	Ag1	70.8(3)
N8	N7	C3	N6	-0.1(7)	O3	Br1	O2	Ag1	-39.4(3)

4.1 CMOF-2

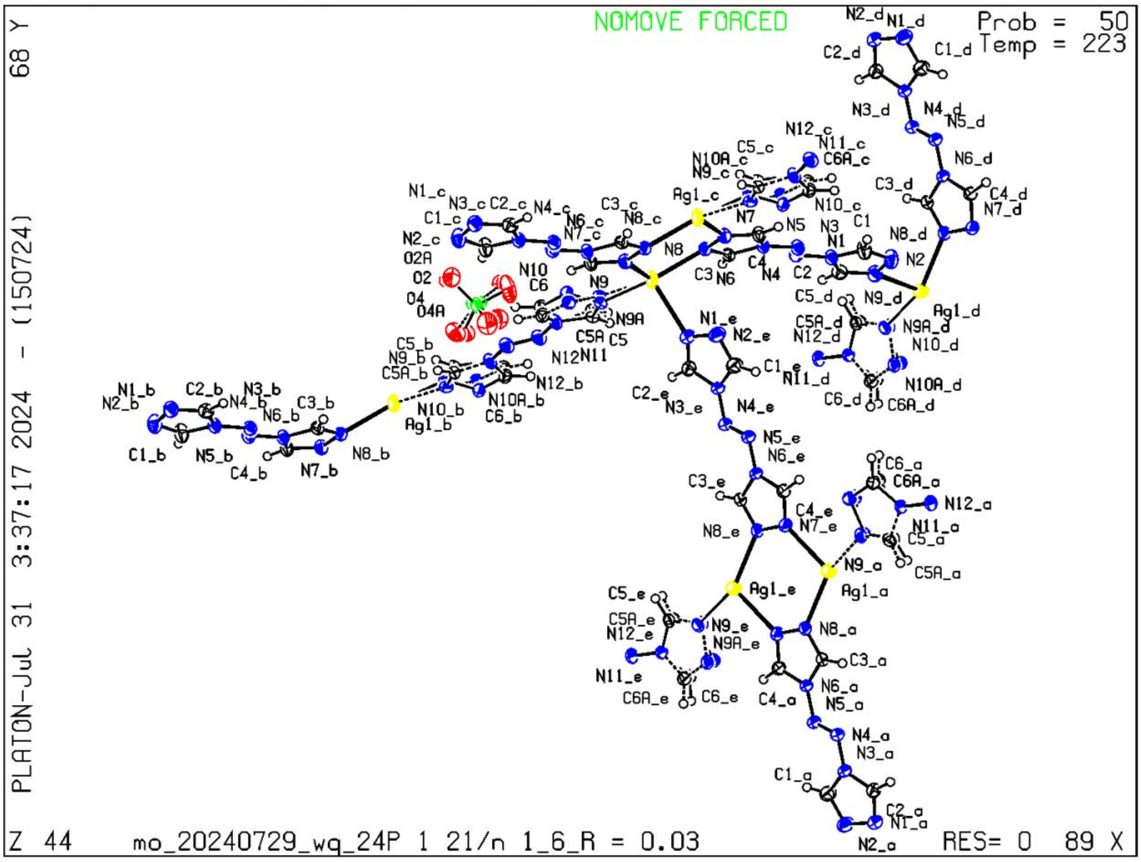

Figure S4. The crystal structure of $[Ag(ATRZ)_{1.5}(ClO_4)]_n$ (CMOF-2, 223K).

Table S5. Crystal data and structure refinement for $[Ag(ATRZ)_{1.5}(ClO_4)]_n$ (CMOF-2, 223 K).

**Compounds**	$[Ag(ATRZ)_{1.5}(ClO_4)]_n$ (CMOF-2)
CCDC number	2383633

Empirical formula	$C_6H_6AgClN_{12}O_4$
Formula weight	453.55
Temperature/K	223.00
Crystal system	monoclinic
Space group	$P2_1/n$
a/Å	5.2924(2)
b/Å	21.1374(8)
c/Å	12.5268(4)
α/°	90
β/°	93.994(2)
γ/°	90
Volume/Å^3	1397.94(9)
Z	4
ρ_{calc}g/cm^3	2.155
μ/mm^{-1}	1.681
F(000)	888.0
Crystal size/mm^3	0.14 × 0.13 × 0.11
Radiation	Mo Kα (λ = 0.71073)
2Θ range for data collection/°	5.048 to 55.016
Index ranges	$-6 \le h \le 6$, $-27 \le k \le 27$, $-16 \le l \le 16$
Reflections collected	30582
Independent reflections	3203 [R_{int} = 0.0910,R_{sigma} = 0.0372]
Data/restraints/parameters	3203/268/300
Goodness-of-fit on F^2	1.074
Final R indexes [I>=2σ (I)]	R_1 = 0.0283, wR_2 = 0.0632
Final R indexes [all data]	R_1 = 0.0417, wR_2 = 0.0679
Largest diff. peak/hole / e Å^{-3}	0.44/-0.69

Table S6. Selected bond lengths [Å] for $[Ag(ATRZ)_{1.5}(ClO_4)]_n$ (CMOF-2, 223 K).

Atom	Atom	Length/Å	Atom	Atom	Length/Å
Ag1	N1[1]	2.445(2)	N11	C5	1.356(9)
Ag1	N7[2]	2.402(2)	N11	C6	1.376(9)
Ag1	N8	2.263(2)	N11	C5A	1.369(10)
Ag1	N9	2.255(8)	N11	C6A	1.378(10)
Ag1	N9A	2.256(9)	N12	N12[3]	1.238(4)
N1	N2	1.401(3)	N9	N10	1.433(11)
N1	C2	1.289(3)	N9	C5	1.292(9)
N2	C1	1.293(4)	N10	C6	1.302(9)
N3	N4	1.383(3)	N9A	N10A	1.431(12)
N3	C1	1.368(3)	N9A	C5A	1.285(10)
N3	C2	1.355(3)	N10A	C6A	1.296(10)
N4	N5	1.241(3)	Cl1	O1	1.413(12)
N5	N6	1.380(3)	Cl1	O2	1.442(12)
N6	C3	1.367(3)	Cl1	O3	1.463(12)
N6	C4	1.368(3)	Cl1	O4	1.424(12)

N7	N8	1.403(3)	Cl1A	O1A	1.415(7)
N7	C4	1.297(3)	Cl1A	O2A	1.429(7)
N8	C3	1.299(3)	Cl1A	O3A	1.430(7)
N11	N12	1.382(3)	Cl1A	O4A	1.448(7)

Table S7. Selected bond Angles [°] for $[Ag(ATRZ)_{1.5}(ClO_4)]_n$ (CMOF-2, 223 K).

Atom	Atom	Atom	Angle/°	Atom	Atom	Atom	Angle/°
N71	Ag1	N12	114.58(8)	C6A	N11	N12	131.0(6)
N8	Ag1	N12	88.36(8)	N124	N12	N11	109.7(3)
N8	Ag1	N71	111.36(7)	N2	C1	N3	110.2(2)
N9	Ag1	N12	98.0(5)	N1	C2	N3	109.9(2)
N9	Ag1	N71	97.7(3)	N8	C3	N6	108.8(2)
N9	Ag1	N8	144.5(5)	N7	C4	N6	109.9(2)
N9A	Ag1	N8	140.2(4)	N10	N9	Ag1	115.7(8)
N2	N1	Ag13	112.33(15)	C5	N9	Ag1	135.1(7)
C2	N1	Ag13	139.63(18)	C5	N9	N10	107.5(8)
C2	N1	N2	107.9(2)	C6	N10	N9	106.4(8)
C1	N2	N1	106.6(2)	N9	C5	N11	109.3(8)
C1	N3	N4	130.2(2)	N10	C6	N11	109.1(8)
C2	N3	N4	124.1(2)	N10A	N9A	Ag1	114.6(9)
C2	N3	C1	105.4(2)	C5A	N9A	Ag1	135.1(8)
N5	N4	N3	108.9(2)	C5A	N9A	N10A	107.6(9)
N4	N5	N6	111.65(19)	C6A	N10A	N9A	106.7(9)
C3	N6	N5	133.8(2)	N9A	C5A	N11	109.4(9)
C3	N6	C4	106.2(2)	N10A	C6A	N11	109.2(9)
C4	N6	N5	119.95(19)	O1	Cl1	O2	108.4(13)
N8	N7	Ag11	125.48(15)	O1	Cl1	O3	107.4(11)
C4	N7	Ag11	127.71(16)	O1	Cl1	O4	118.9(13)
C4	N7	N8	106.8(2)	O2	Cl1	O3	105.1(13)
N7	N8	Ag1	117.25(15)	O4	Cl1	O2	108.7(12)
C3	N8	Ag1	127.28(16)	O4	Cl1	O3	107.5(14)
C3	N8	N7	108.4(2)	O1A	Cl1A	O2A	110.7(8)
C5	N11	N12	122.5(5)	O1A	Cl1A	O3A	110.0(5)
C5	N11	C6	107.0(7)	O1A	Cl1A	O4A	105.4(9)
C6	N11	N12	130.4(5)	O2A	Cl1A	O3A	110.2(7)
C5A	N11	N12	122.6(6)	O2A	Cl1A	O4A	110.0(7)
C5A	N11	C6A	106.4(8)	O3A	Cl1A	O4A	110.4(8)

Table S8. Selected torsion Angles [°] for $[Ag(ATRZ)_{1.5}(ClO_4)]_n$ (CMOF-2, 223 K).

Atom	Atom	Atom	Atom	Angle/°	Atom	Atom	Atom	Atom	Angle/°
Ag11	N1	N2	C1	177.5(2)	N12	N11	C6A	N10A	175.4(7)
Ag11	N1	C2	N3	-176.2(2)	C1	N3	N4	N5	-7.0(4)
Ag12	N7	N8	Ag1	-29.5(2)	C1	N3	C2	N1	0.3(3)

Ag12	N7	N8	C3	177.71(17)	C2	N1	N2	C1	0.6(3)
Ag12	N7	C4	N6	-177.19(17)	C2	N3	N4	N5	166.2(2)
Ag1	N8	C3	N6	-149.61(17)	C2	N3	C1	N2	0.1(3)
Ag1	N9	N10	C6	176.2(10)	C3	N6	C4	N7	-0.9(3)
Ag1	N9	C5	N11	-171.9(17)	C4	N6	C3	N8	0.7(3)
Ag1	N9A	N10A	C6A	-173.5(10)	C4	N7	N8	Ag1	152.59(18)
Ag1	N9A	C5A	N11	166.8(18)	C4	N7	N8	C3	-0.2(3)
N1	N2	C1	N3	-0.4(3)	N9	N10	C6	N11	-6.0(14)
N2	N1	C2	N3	-0.5(3)	N10	N9	C5	N11	-8.2(17)
N3	N4	N5	N6	-178.1(2)	C5	N11	N12	N123	166.6(11)
N4	N3	C1	N2	174.3(3)	C5	N11	C6	N10	1.2(12)
N4	N3	C2	N1	-174.4(2)	C5	N9	N10	C6	8.9(17)
N4	N5	N6	C3	10.3(4)	C6	N11	N12	N123	-17.2(12)
N4	N5	N6	C4	-173.5(2)	C6	N11	C5	N9	4.7(14)
N5	N6	C3	N8	177.3(3)	N9A	N10A	C6A	N11	7.5(16)
N5	N6	C4	N7	-178.0(2)	N10A	N9A	C5A	N11	7.4(18)
N7	N8	C3	N6	-0.3(3)	C5A	N11	N12	N123	179.6(12)
N8	N7	C4	N6	0.7(3)	C5A	N11	C6A	N10A	-3.2(14)
N12	N11	C5	N9	-178.4(8)	C5A	N9A	N10A	C6A	-9.4(19)
N12	N11	C6	N10	-175.4(7)	C6A	N11	N12	N123	1.2(13)
N12	N11	C5A	N9A	178.3(9)	C6A	N11	C5A	N9A	-3.0(16)

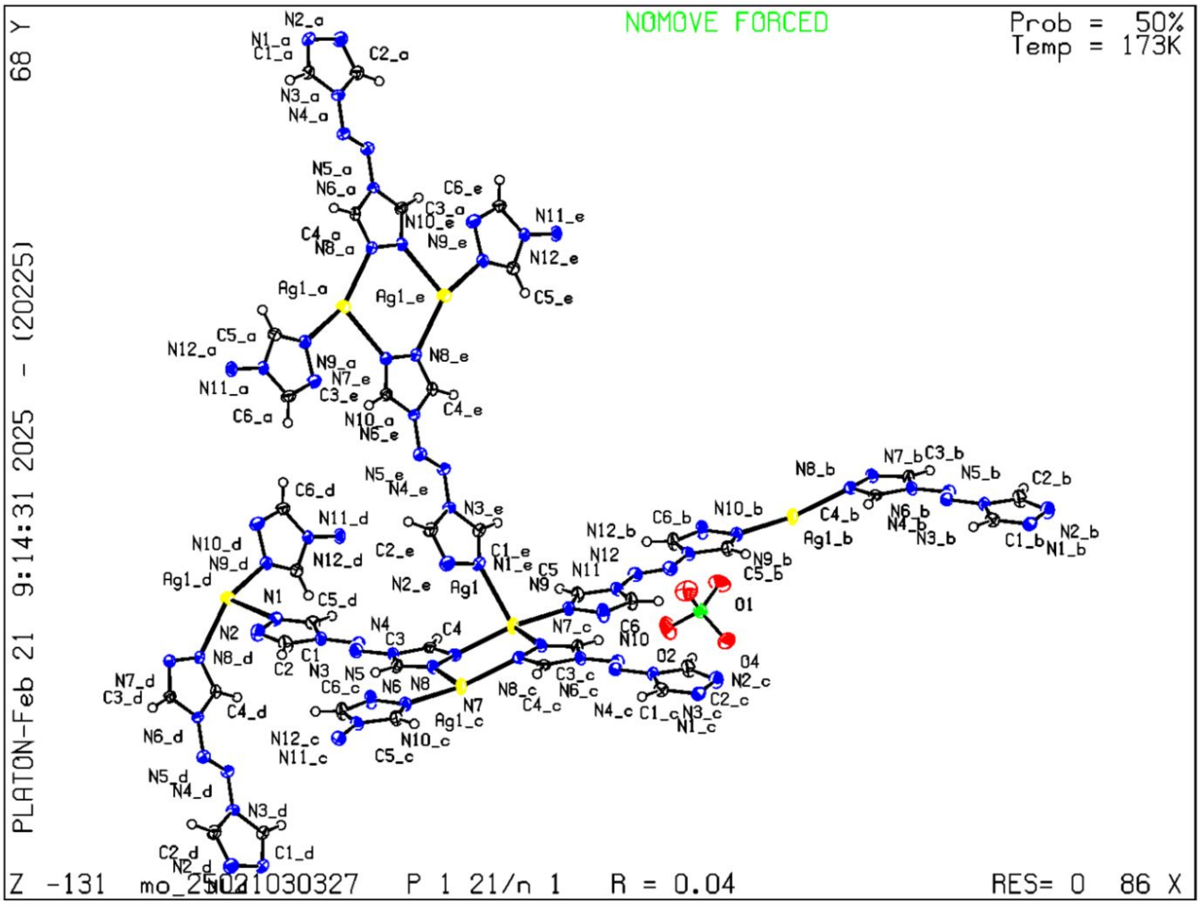

Figure S5. The crystal structure of $[Ag(ATRZ)_{1.5}(ClO_4)]_n$ (CMOF-2, 173K).

Table S9. Crystal data and structure refinement for $[Ag(ATRZ)_{1.5}(ClO_4)]_n$ (CMOF-2, 173 K).

**Compounds**	$[Ag(ATRZ)_{1.5}(ClO_4)]_n$ (CMOF-2)
Empirical formula	$C_6H_6AgClN_{12}O_4$
Formula weight	453.55
Temperature/K	173.00
Crystal system	monoclinic
Space group	$P2_1/n$
a/Å	5.2470(3)
b/Å	21.2344(9)
c/Å	12.4319(6)
α/°	90
β/°	93.195(2)
γ/°	90
Volume/Å^3	1382.97(12)
Z	4
ρ_{calc}g/cm^3	2.178
μ/mm^{-1}	1.700
F(000)	888.0
Crystal size/mm^3	0.1 × 0.08 × 0.06
Radiation	MoKα (λ = 0.71073)
2Θ range for data collection/°	5.048 to 52.108
Index ranges	$-6 \le h \le 6$, $-26 \le k \le 26$, $-15 \le l \le 15$
Reflections collected	17496
Independent reflections	2737 [R_{int} = 0.0917, R_{sigma} = 0.0490]
Data/restraints/parameters	2737/0/217
Goodness-of-fit on F^2	1.052
Final R indexes [I>=2σ (I)]	R_1 = 0.0364, wR_2 = 0.0670
Final R indexes [all data]	R_1 = 0.0474, wR_2 = 0.0723
Largest diff. peak/hole / e Å^{-3}	0.48/-0.62

Table S10. Selected bond lengths [Å] for $[Ag(ATRZ)_{1.5}(ClO_4)]_n$ (CMOF-2, 173 K).

**Atom**	**Atom**	**Length/Å**	**Atom**	**Atom**	**Length/Å**
Ag1	N1^1	2.433(3)	N7	Ag1^2	2.393(3)
Ag1	N7^2	2.393(3)	N7	N8	1.398(4)
Ag1	N8	2.260(3)	N7	C3	1.298(5)
Ag1	N9	2.251(3)	N8	C4	1.305(5)
N1	Ag1^3	2.433(3)	N9	N10	1.396(5)
N1	N2	1.401(5)	N9	C5	1.296(5)
N1	C1	1.296(5)	N10	C6	1.298(5)
N2	C2	1.297(5)	N11	N12	1.380(4)
N3	N4	1.387(4)	N11	C5	1.359(5)
N3	C1	1.363(5)	N11	C6	1.367(5)
N3	C2	1.366(5)	N12	N12^4	1.233(7)
N4	N5	1.234(5)	Cl1	O1	1.429(3)

N5	N6	1.394(4)	Cl1	O2	1.427(3)
N6	C3	1.359(5)	Cl1	O3	1.431(3)
N6	C4	1.361(5)	Cl1	O4	1.428(3)

Table S11. Selected bond Angles [°] for $[Ag(ATRZ)_{1.5}(ClO_4)]_n$ (CMOF-2, 173 K).

Atom	Atom	Atom	Angle/°	Atom	Atom	Atom	Angle/°
N7[1]	Ag1	N1[2]	115.60(12)	C4	N8	Ag1	127.0(3)
N8	Ag1	N1[2]	87.78(12)	C4	N8	N7	108.1(3)
N8	Ag1	N7[1]	111.67(11)	N10	N9	Ag1	117.0(2)
N9	Ag1	N1[2]	100.17(12)	C5	N9	Ag1	135.4(3)
N9	Ag1	N7[1]	97.82(11)	C5	N9	N10	107.7(3)
N9	Ag1	N8	142.54(12)	C6	N10	N9	107.5(3)
N2	N1	Ag1[3]	112.2(2)	C5	N11	N12	122.7(3)
C1	N1	Ag1[3]	140.1(3)	C5	N11	C6	106.2(3)
C1	N1	N2	107.6(3)	C6	N11	N12	131.1(3)
C2	N2	N1	107.1(3)	N124	N12	N11	109.4(4)
C1	N3	N4	123.5(3)	N1	C1	N3	109.7(4)
C1	N3	C2	105.7(3)	N2	C2	N3	109.9(4)
C2	N3	N4	130.6(3)	N7	C3	N6	109.6(4)
N5	N4	N3	108.3(3)	N8	C4	N6	108.5(3)
N4	N5	N6	111.3(3)	N9	C5	N11	109.4(4)
C3	N6	N5	119.6(3)	N10	C6	N11	109.2(4)
C3	N6	C4	106.8(3)	O1	Cl1	O3	109.1(2)
C4	N6	N5	133.6(3)	O2	Cl1	O1	110.3(2)
N8	N7	Ag1[1]	125.0(2)	O2	Cl1	O3	110.0(2)
C3	N7	Ag1[1]	128.0(3)	O2	Cl1	O4	108.6(2)
C3	N7	N8	107.0(3)	O4	Cl1	O1	110.0(2)
N7	N8	Ag1	117.6(2)	O4	Cl1	O3	108.7(2)

Table S12. Selected torsion Angles [°] for $[Ag(ATRZ)_{1.5}(ClO_4)]_n$ (CMOF-2, 173 K).

Atom	Atom	Atom	Atom	Angle/°	Atom	Atom	Atom	Atom	Angle/°
Ag1[1]	N1	C1	N3	-176.4(3)	N10	N9	C5	N11	-0.5(5)
Ag1[2]	N7	N8	Ag1	-29.1(4)	N12	N11	C5	N9	-179.9(3)
Ag1[2]	N7	N8	C4	178.5(3)	N12	N11	C6	N10	-179.9(4)
Ag1[2]	N7	C3	N6	-178.0(2)	C1	N1	N2	C2	0.4(5)
Ag1	N8	C4	N6	-149.3(3)	C1	N3	N4	N5	166.3(4)
Ag1	N9	N10	C6	-179.8(3)	C1	N3	C2	N2	0.2(5)
Ag1	N9	C5	N11	179.5(3)	C2	N3	N4	N5	-6.6(6)
N1	N2	C2	N3	-0.4(5)	C2	N3	C1	N1	0.1(5)
N2	N1	C1	N3	-0.3(5)	C3	N6	C4	N8	0.8(4)
N3	N4	N5	N6	-178.1(3)	C3	N7	N8	Ag1	151.9(3)
N4	N3	C1	N1	-174.3(3)	C3	N7	N8	C4	-0.6(4)
N4	N3	C2	N2	174.0(4)	C4	N6	C3	N7	-1.1(5)

N4	N5	N6	C3	-173.7(4)	C5	N9	N10	C6	0.2(5)
N4	N5	N6	C4	9.5(6)	C5	N11	N12	N12^{3}	172.5(4)
N5	N6	C3	N7	-178.8(3)	C5	N11	C6	N10	-0.5(5)
N5	N6	C4	N8	177.9(4)	C6	N11	N12	N12^{3}	-8.2(7)
N7	N8	C4	N6	-0.2(4)	C6	N11	C5	N9	0.6(5)
N8	N7	C3	N6	1.0(4)					
